# The Impact of Smoking on Clinical Outcomes after Percutaneous Coronary Intervention in Women Compared to Men

**DOI:** 10.1155/2021/6619503

**Published:** 2021-03-16

**Authors:** Seyyed Saeed Mohammadi, Mohammad Javad Zibaeenezhad, Mehrab Sayadi, Soorena Khorshidi, Ehsan Hadiyan, Iman Razeghian-Jahromi

**Affiliations:** Cardiovascular Research Center, Shiraz University of Medical Sciences, Shiraz, Iran

## Abstract

**Background:**

For decades, cardiovascular diseases (CVD) have been known as men's disease. However, recent research studies showed that they have become more common in women. Smoking is a strong risk factor for CVD especially that of coronary artery disease (CAD). Several studies reported that women are more susceptible to drastic sequels of smoking than men. There is limited data regarding the impact of smoking on post-revascularization clinical events stratified by gender. This study aimed to investigate if gender significantly changes the incidence of adverse clinical outcomes after percutaneous coronary intervention (PCI) among those with history of smoking.

**Methods:**

Participants were selected from two hospitals from 2003 to 2019. Among patients who had PCI (index PCI), those with stable CAD who underwent elective PCI were included. Exclusion criteria were defined as primary PCI and those with multiple prior revascularizations. Participants were followed up seeking for major adverse cardiac events (MACE) including revascularization (PCI or coronary artery bypass grafting), myocardial infarction, and coronary death in three time intervals according to the time of index PCI (short term: up to 24 hours, mid-term: 24 hours to less than 6 months, and long term: more than 6 months).

**Results:**

Of the 1799 patients, 61% were men and 47.08% had history of smoking (75% of the smokers were men). At the time of index PCI, smokers were significantly younger than nonsmokers. Also, MACE were significantly higher in smokers than nonsmokers, which was particularly pronounced at the long-term interval. In the nonsmokers group, there was no difference in MACE occurrence between men and women. However, of the smokers, women showed significantly higher MACE rate compared with men peers.

**Conclusion:**

Smoking makes women more prone to MACE in comparison to men among patients with stable CAD after PCI with drug-eluting stent.

## 1. Introduction

Coronary artery disease (CAD) has become the pre-dominant cause of mortality and morbidity throughout the world [1]. Some investigations reported that the occurrence of cardiovascular diseases (CVD) is more common in women [2] and cardiovascular ischemia is one of the primary contributors to illness and lethality in this gender [3]. Meanwhile, smoking has been considered as an established risk factor for CAD [4]. The risk of MI in women was reported to be twofold higher compared with men due to smoking [3]. Also, women were shown to be at 25% greater risk for CAD than men with the same smoking degree [5].

Smoking augmented the incidence of adverse events after percutaneous coronary intervention (PCI) [6, 7]. Reviewing the literature, limited data is available on comparison of clinical events between men and women after revascularization. The objective of our study is to investigate the association of gender with post-PCI major adverse cardiac events (MACE) in three time intervals in patients with history of smoking.

## 2. Materials and Methods

Between 2003 and 2019, patients who had PCI (index PCI) in two hospitals were considered. Inclusion criteria were elective PCI, i.e., patients with stable CAD. Patients who had PCI due to acute MI (primary PCI) and those with multiple PCI in the past were excluded as well as lost-to-follow-up participants. If anatomy of the coronary arteries was suitable, PCI was performed. The criteria of doing angioplasty were based on ≥75% narrowing in left anterior descending artery, diagonal, left circumflex artery, obtuse marginal branches, right coronary artery, posterior descending artery, or posterior left ventricular branch. At the time of index PCI, heparin (80–100 mg/kg) was administered. The preload of 600 mg clopidogrel and 325 mg aspirin (ASA) were given followed by 150 mg clopidogrel and 325 mg ASA for three weeks. It was continued by 75 mg/day clopidogrel and 80 mg ASA (160 mg in diabetics) in all the patients at least for two years. In addition, atorvastatin (40–80 mg daily) was given to all the patients. Drug-eluting stent (DES) from first generation (Sirolimus Eluting Stent, SES; Paclitaxel Eluting Stent, PES) and second generation (Zotarolimus Eluting Stent, ZES; Everolimus Eluting Stent, EES) was used in all of the participants, based on their availability.

History of smoking is defined as being a smoker of any amount of any type (cigarette, water pipe, cigar, and pipe) before index PCI. Patients were considered hyperlipidemic if they had hyperlipidemia (HLP) or used antihyperlipidemic medications [8]. Patients who had fasting plasma glucose of ≥126 mg/dL (7.0 mmol/L) or 2 h plasma glucose of ≥200 mg/dL (11.1 mmol/L) during oral glucose tolerance test or A1C of ≥6.5% (48 mmol/mol) or with classic symptoms of hyperglycemia or hyperglycemic crisis, a random plasma glucose of ≥200 mg/dL (11.1 mmol/L), or receiving antihyperglycemic medications before index PCI were considered as having diabetes mellitus (DM). Patients who had systolic blood pressure of ≥130 mmHg or diastolic blood pressure of ≥80 mmHg or both or receiving antihypertensive medications before index PCI were considered as having hypertension (HTN) [9].

Occurrence of MACE (coronary death, myocardial infarction (MI), and revascularization including PCI or coronary artery bypass grafting) was sought in the follow-up phase. During hospitalization, data regarding incidence of MACE could be easily collected. Upon discharge, the patients were asked to adhere to a periodic visiting schedule at defined clinic. Otherwise, phone calls were made looking for incidence of MACE. MACE was categorized with respect to the time of index PCI into three intervals. First 24 hours following the index PCI was considered short-term, between 24 hours and less than 6 months and equal to or more than 6 months were defined as mid- and long-term, respectively. Age means the age of the patients at the time of index PCI. Time-to-event is the time duration from index PCI to the incidence of first MACE.

Categorical and continuous variables are presented as number (%) and mean ± standard deviation (SD), respectively. Statistical tests (chi-square, independent sample *t*-test, and cox proportional-hazards regression) were used for analyses with *P* value of less than 0.05 showing significant difference. All the analyses were performed using the Statistical Package for Social Sciences version 22.0 (SPSS Inc., Chicago, IL, USA).

## 3. Results

The mean age of the participants at the time of index PCI was 60.14 ± 11.21 years. Women were older (62.38 ± 10.34) than men (58.72 ± 11.51) (*P* < 0.001). Among men, smokers were younger (56.82 ± 11.27) than nonsmokers (61.30 ± 11.32) (*P* < 0.001) while there was no difference between smokers (61.34 ± 11.00) and nonsmokers (62.84 ± 10.01) in women (*P*=0.090). Men to women ratio was 1.55. Minimum and maximum clinical follow-up duration were 10 days and 201.47 months with a median of 66.5 ± 10.66 months. Minimum and maximum time-to-event were 15 days and 150.6 months with a median of 51.47 ± 44.80 months.

Baseline characteristics of the population stratified by smoking status are demonstrated in Table 1. In total, 47.08% of the subjects were smokers with dominance of men (nearly 75%). Smokers were significantly younger (57.95 ± 11.37) than nonsmokers (60.08 ± 10.70) at the time of index PCI. Unlike HTN that was higher in the nonsmokers, the prevalence of DM and HLP was not different between smokers and nonsmokers. MACE incidence was significantly higher in smokers after nonsmoker peers (*P* < 0.001). No difference was seen in time-to-event between smoker and nonsmoker patients. Also, there were no significant differences regarding type of MACE (death, MI, and target vessel revascularization) between smoker (28.8%, 6%, 65%) and nonsmoker (18%, 6%, 76%) groups.

MACE incidence was 7.2% (130 patients) with no difference between men and women patients (Table 2). Comparing the two groups (MACE and not-MACE) showed that smokers were significantly outnumbered in the MACE group while other variables including age at the time of index PCI, gender, DM, HTN, and HLP were not different.

**Figure 1:**
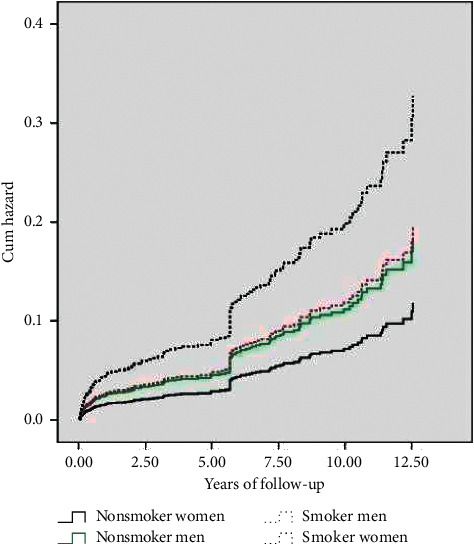
Hazard rate for interaction of sex and smoking. After adjusting other risk factors including age, hDM, hHTN, and hHLP, female patients with smoking remained at higher risk than male patients with smoking. HR for female nonsmokers, male nonsmokers, and male smokers versus female smokers were (0.360, CI: 0.203–0.637), (0.562 CI: 0.337–0.937), and (0.597 CI: 0.376–0.948), respectively.

In Table 3, joint effect of smoking with gender and other risk factors on the incidence of MACE was delineated. When we added the effect of smoking to the effect of female gender, MACE incidence was statistically increased. Neither male gender nor other risk factors changed MACE significantly when combined with smoking. Hazard rate for interaction of gender and smoking is shown in Figure 1. It shows that, after adjusting other variables (age, DM, HTN, and HLP), smoker women were at a higher risk for MACE incidence compared with smoker men. We compared the incidence of MACE in short, mid-, and long term (Table 4). None of the participants experienced MACE in short-term interval. Of the 33 MACEs in mid-term, 22 occurred in smoker group which was significantly higher than the nonsmokers (*P* < 0.038). The remaining 97 MACEs (74.61%) happened in the long-term interval with 58 of them in smokers (significantly higher compared with nonsmokers, *P*=0.006). Table 4 also demonstrates that MACE incidence was statistically higher in smoker women in comparison to nonsmoker peers in both mid- and long-term intervals. Indeed, it seems that smoking significantly increased MACE in women compared with men at long-term interval as well as in total duration of follow-up period.

## 4. Discussion

The main findings that resonate from the present study are as follows. (1) About half of the CAD patients who underwent elective PCI had history of smoking. (2) Smokers were younger at the time of index PCI than nonsmokers. (3) Smoking was a stronger predictor of MACE than age, gender, DM, HTN, and HLP. (4) Smoker women were significantly more prone to MACE incidence than nonsmoker women while no difference was observed between smoker and nonsmoker men in this regard. Although nearly 75% of the smokers were men, MACE incidence was significantly higher in the smoker women.

There are debates in the pathophysiology of cardiovascular consequences of smoking [10–14]. Smoking promotes the generation of free radicals through increasing the oxidative stress and decreasing the protecting nitric oxide [15, 16]. These changes tip the cellular balance towards a pro-atherogenic and pro-thrombotic milieu. Also, smoking induces vasomotor dysfunction followed by impairment in lipid homeostasis and thereby facilitates the progression of inflammation at vascular and systemic scales [11].

Free radical-mediated oxidative stress is known as the pivotal reason for the development of atherosclerosis especially in women [17]. During menopause, hormonal changes lead to cholesterol oxidation, which provides a substrate for atherosclerosis development. Also, smoking instigates severe stress responses in the female gender [18]. The greater risk for coronary heart disease in women than men is possibly attributed to higher absorption of toxic chemicals in women as well [19].

Smoking induces platelet hyperactivity reducing the responsiveness of antiplatelet drugs like aspirin in patients with stable CAD [20]. A prospective cohort study stated that epigenetic alterations like the degree of methylation in a gene locus was associated with mortality in a population of stable CAD [21]. This gene locus expresses a protein with roles in platelet activation, intimal hyperplasia, and inflammation [22]. Smoking habit changes the methylation level [21].

Active smoking was shown to be in a consistent association with poor outcomes among patients with stable CAD [23]. Even light smokers experienced more cardiovascular and all-cause mortality compared with nonsmokers in such patients [24–26]. Cardiovascular risk factors are also severe in the former smokers, albeit lower than active peers [23]. This shows that smoking imposes harmful effects at any level. One important point that should be interpreted with caution is that, unlike some cardiovascular risk factors such as DM, HTN, and HLP that retain their deleterious effects until diagnosis and treatment, smoking is completely an extrinsic factor and its harms depend on the duration of smoking [27].

In an investigation, smoking was characterized as the only risk factor for MACE in long-term clinical follow-up, which is in line with the findings of the present study. However, association of history of smoking with mortality was ruled out in the other study [28]. Although the prevalence of smoking has been reported to be five times higher in men compared with women [29], the risk of MI due to smoking in men is half that of women [30]. Indeed, the prevalence of other CAD risk factors such as HTN and DM was lower in women, but in-hospital mortality and MACE were reported high in this gender, even irrespective of smoking status (current or former) [28]. Another possible explanation is that CAD is often underdiagnosed or misdiagnosed in women and hence they present at later stages with probably a more complicated condition. To substantiate, smoker women with 25% greater risk for coronary heart disease lose more years of life than smoker men [31].

A conflicting belief that was reported in seminal studies claim that smoking is associated with a lower rate of subsequent targeted lesion revascularization in patients with PCI. This so-called smoker's paradox means there is no superiority of being nonsmoker with respect to the incidence of adverse events in CAD patients [8, 32, 33]. MACE incidence was nearly double in the smoker group compared with nonsmoker peers in our study reinforcing imperfection of the smoker's paradox theory once more.

Data on clinical outcomes after revascularization were scarce especially considering gender differences [34, 35]. The majority of studies discussed the effect of smoking on the incidence of outcomes in PCI setting, but they did not consider the used stents at all or they considered bare metal stents only. Therefore, there is a need to provide insights from the area of PCI with DES stratified by gender. In our population of stable CAD with DES implantation during PCI, elevated occurrence of MACE in the smoker women compared with all other groups was evidenced in our study. This is in line with other findings stating that the female gender possesses a significant contribution to adverse cardiovascular events [36]. The advantages of our study were high number of participants and long period of follow-up (median of 66.5 ± 10.66 months).

Among the limitations of the study is that the menstrual cycle of women patients was not recorded. Phases of the menstrual cycle affect the incidence of cardiovascular-related events. However, as the median age of the women was about 62 years, it is anticipated that all of them were in the menopause phase. Evaluation of the smoking status of the participants was done in a self-reporting context. This may lead to some inaccuracies in terms of desire to be nonsmoker. Also, some participants may have changed smoking habit in the follow-up phase especially after the occurrence of a clinical event.

## 5. Conclusion

In patients with stable CAD, smokers suffered more MACE than nonsmokers in the post-PCI setting. Although the number of men was higher and smoking was more prevalent in this gender, women experienced more MACE. This shows the much more vulnerability of women due to smoking.

## Figures and Tables

**Table 1:** Baseline characteristics of 1799 participants stratified by smoking status.

	Smoker (*n* = 847)	Nonsmoker (*n* = 952)	*P* ^+^	OR	95% CI for OR		*P*
					Lower	Upper	
Age (months)	57.95 ± 11.37	60.08 ± 10.70	<**0**.**001**	0.976	0.967	0.985	<**0**.**001**
Gender (male)	634 (74.9%)	467 (49.1%)	<**0**.**001**	2.586	2.088	3.204	<**0**.**001**
DM	231 (27.3%)	302 (31.7%)	**0.039**	0.988	0.786	1.243	0.918
HTN	385 (45.5%)	614 (64.5%)	<**0**.**001**	0.643	0.521	0.794	<**0**.**001**
HLP	398 (47.0%)	519 (54.5%)	**0.001**	1.015	0.818	1.258	0.894
MACE	80 (9.4%)	50 (5.3%)	**0.001**	1.878	1.276	2.764	**0.001**
Time-to-event (months)	50.79 ± 45.33	52.56 ± 44.37	0.828	—	—	—	—

DM: diabetes mellitus, HTN: hypertension, HLP: hyperlipidemia, MACE: major adverse cardiac events, *n*: number, *P*^+^: extracted from *t*-test or chi-square test in univariate approach, OR: odds ratio (adjusted), CI: confidence interval, and *P*: extracted from multiple logistic regression analysis. Bold values imply significant differences.

**Table 2:** Comparison of different variables between MACE and not-MACE groups.

	MACE (*n* = 130)	Not-MACE (*n* = 1669)	*P* ^+^	HR	95% CI for HR		*P*
					Lower	Upper	
Age (months)	57.74 ± 10.90	60.33 ± 11.21	**0.011**	0.985	0.969	1.001	0.071
Gender (male)	81 (62.3%)	1020 (61.1%)	0.788	1.058	0.718	1.560	0.774
DM	44 (33.8%)	489 (29.3%)	0.274	1.431	0.967	2.118	0.073
HTN	73 (56.2%)	926 (55.5%)	0.882	1.290	0.882	1.884	0.189
HLP	62 (47.7%)	855 (51.2%)	0.437	0.742	0.508	1.085	0.124
Smoking	80 (61.5%)	767 (46.0%)	**0.001**	1.617	1.116	2.343	**0.011**

DM: diabetes mellitus, HTN: hypertension, HLP: hyperlipidemia, MACE: major adverse cardiac events, *P*^+^: extracted from *t*-test or chi-square test in univariate approach, HR: hazard ratio (adjusted), CI: confidence interval, *n* = number, HR: adjusted HR, and *P*: extracted from multiple proportional hazard cox regression analysis. Bold values imply significant differences.

**Table 3:** Joint effect of smoking with gender and other risk factors.

	MACE	Not-MACE	HR (95% CI)	*P*
Smoker (*n*, %)	80 (61.5%)	767 (46%)	1.27 (0.80–2.01)	0.300
Female (smoker/nonsmoker)	29 (22.3%)	184 (11%)	1.86 (1.23–2.82)	**0.003**
Male (smoker/nonsmoker)	51 (39.2)	583 (34.9)	1.12 (0.78–1.59)	0.527
Female (DM/non-DM)	21 (42.9)	251 (38.7)	1.38 (0.78–2.43)	0.262
Male (DM/non-DM)	23 (28.4)	238 (23.3)	1.34 (0.82–2.17)	0.230
Female (HTN/non-HTN)	35 (71.4)	478 (73.7)	0.96 (0.51–1.79)	0.909
Male (HTN/non-HTN)	38 (46.9)	448 (43.9)	1.17 (0.75–1.81)	0.470
Female (HLP/non-HLP)	31 (63.3)	423 (65.2)	0.92 (0.52–1.66)	0.804
Male (HLP/non-HLP)	31 (38.3)	432 (42.4)	0.83 (0.53–1.31)	0.440

MACE: major adverse cardiac events, HR: hazard ratio, CI: confidence interval, DM: diabetes mellitus, HTN: hypertension, and HLP: hyperlipidemia. Data are presented as number (%). Bold values imply significant differences.

**Table 4:** MACE incidence in mid-tem, long term, and total between smokers and nonsmokers.

		Smokers (*n* = 847)	Nonsmokers (*n* = 952)	*P*
Mid-term MACE	Male	14/634 (2.2%)	7/467 (1.5%)	0.395
	Female	8/213 (3.8%)	4/485 (1.0%)	**0.028**
	*P*	0.219	0.518	

Long-term MACE	Male	37/565 (6.5%)	23/403 (5.7%)	0.592
	Female	21/188 (11.2%)	16/434 (3.7%)	<**0**.**001**
	*P*	**0.040**	0.166	

MACE	Male	51/634 (8.0%)	30/467 (6.4%)	0.309
	Female	29/213 (13.6%)	20/485 (4.1%)	<**0**.**001**
	*P*	**0.016**	0.112	

Mid-term: 24 hours up to 6 months after index PCI, long term: 6 months after index PCI, MACE: major adverse cardiac event, and *n* = number. Bold values imply significant differences.

## Data Availability

The data used to support the findings of this study are available from the corresponding author upon request.
